# Inflammatory Mediators Suppress FGFR2 Expression in Human Keratinocytes to Promote Inflammation

**DOI:** 10.1080/10985549.2024.2399766

**Published:** 2024-09-28

**Authors:** Luca Ferrarese, Michael Koch, Artemis Baumann, Liliana Bento-Lopes, Daria Wüst, Ivan Berest, Manfred Kopf, Sabine Werner

**Affiliations:** Institute of Molecular Health Sciences, Department of Biology, ETH Zürich, Zürich, Switzerland

**Keywords:** Atopic dermatitis, FGF, inflammation, keratinocyte, signaling

## Abstract

Fibroblast growth factors (FGFs) are key orchestrators of development, tissue homeostasis and repair. FGF receptor (FGFR) deficiency in mouse keratinocytes causes an inflammatory skin phenotype with similarities to atopic dermatitis, but the human relevance is unclear. Therefore, we generated human keratinocytes with a CRISPR/Cas9-induced knockout of *FGFR2*. Loss of this receptor promoted the expression of interferon-stimulated genes and pro-inflammatory cytokines under homeostatic conditions and in particular in response to different inflammatory mediators. Expression of FGFR2 itself was strongly downregulated in cultured human keratinocytes exposed to various pro-inflammatory stimuli. This is relevant *in vivo*, because bioinformatics analysis of bulk and single-cell RNA-seq data showed strongly reduced expression of *FGFR2* in lesional skin of atopic dermatitis patients, which likely aggravates the inflammatory phenotype. These results reveal a key function of FGFR2 in human keratinocytes in the suppression of inflammation and suggest a role of FGFR2 downregulation in the pathogenesis of atopic dermatitis and possibly other inflammatory diseases.

## Introduction

FGFs and their high affinity tyrosine kinase receptors (FGFR1-FGFR4) play key roles in development, homeostasis and repair of different tissues and organs, and their abnormal expression or activity is associated with a broad variety of human diseases and impairments in tissue repair.^1–3^ FGFs constitute a large and highly conserved family of signaling molecules, denoted FGF1 – FGF23. Of particular importance for epithelial tissues are FGF7 and FGF10. They predominantly (FGF10) or even exclusively (FGF7) activate the 3b splice variant of FGFR2, called FGFR2b.^3–5^ The latter is generated by alternative splicing in the third immunoglobulin-like domain in the extracellular part of the receptor, which regulates the FGFR2 binding specificities.^3^^,^^4^ Binding of FGFs to their receptors triggers activation of three principal intracellular signaling pathways: the mitogen-activated protein kinase (MAPK) pathway, the phosphoinositide 3-kinase (PI3K) pathway, and the phospholipase Cγ (PLCγ) pathway.^3^^,^^4^

Activation of FGFR signaling in keratinocytes via FGF7 (also known as keratinocyte growth factor (KGF)) and the related FGF10 induces cell proliferation and migration and also affects the differentiation process.^6–8^ In addition, these FGFs have strong cytoprotective activities under stress conditions.^9–11^ Therefore, efforts have been made to translate these findings into clinical applications, particularly in situations where protection or rapid regeneration of epithelial tissues is required. A recombinant, truncated form of FGF7, known as palifermin, is approved for use as supportive care treatment to prevent and manage severe mucositis in cancer patients undergoing radio- and/or chemotherapy.^10^^,^^12^ The beneficial effect of FGF7 is thought to result from its effect on the detoxification of reactive oxygen species and from its direct anti-apoptotic activity,^13^ but additional mechanisms cannot be excluded.

To study the role of endogenous FGF7 and FGF10 in vivo, we previously generated mice lacking functional *Fgfr1* and *Fgfr2* genes in keratinocytes. They displayed delayed wound healing and showed several characteristic features of atopic dermatitis (AD) even in the absence of injury, including keratinocyte hyperproliferation, an impaired epidermal barrier due to diminished expression of tight junction components, overexpression of pro-inflammatory cytokines and of interferon-stimulated genes (ISGs) and an immune cell infiltrate similar to the one observed in AD.^14^^,^^15^ This phenotype was mainly a consequence of the Fgfr2 deficiency, but the additional loss of Fgfr1 strongly aggravated the phenotype because of partial redundancy of these receptors. By contrast, additional loss of Fgfr3 had only a very minor effect.^16^ Together, these data strongly suggest that Fgfr2 is primarily responsible for the control of barrier function and inflammation in the murine epidermis. However, the role of endogenous FGFR2 in human keratinocytes in the control of skin inflammation is largely unknown. To address this question, we generated FGFR2-deficient human keratinocytes. We show the usefulness of this model to study FGF signaling in keratinocytes, and we describe a regulatory loop, which controls skin inflammation via keratinocytes. Most importantly, our findings suggest an unexpected role of reduced FGF signaling in the pathogenesis of AD.

## Results

### Expression of FGF receptors in human primary keratinocytes (HPK) and HaCaT cells

To determine the function of FGFR1 and FGFR2 in human keratinocytes, we aimed to generate cells lacking individual receptors. Because HPK undergo replicative senescence upon clonal expansion, we first tested if HaCaT cells are suitable for this purpose. This spontaneously immortalized, but nontumorigenic keratinocyte cell line retains key features of HPK,^17^ including a similar response to FGF7.^18^

**Figure 1.**
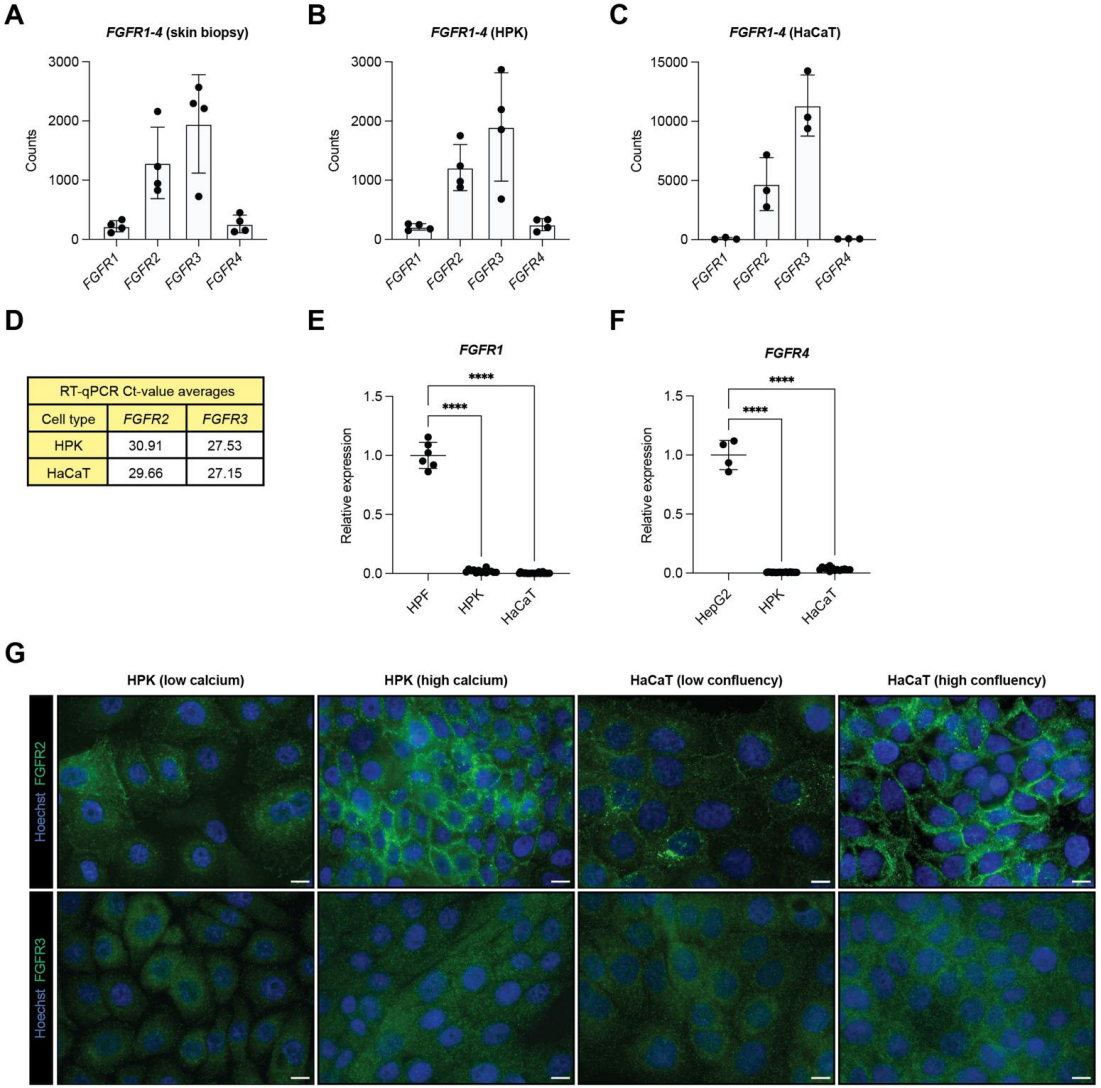
FGFR2 and FGFR3 are the predominant FGF receptors in human keratinocytes. (A, B, C) *FGFR1*, *FGFR2*, *FGFR3* and *FGFR4* expression counts in RNA-seq data from (A) full-thickness human skin biopsies (GSE171170) (N = 4 donors), (B) human primary keratinocytes (HPK) (GSE171170) (N = 4 donors) and (C) HaCaT cells (GSE267530) (N = 3 clonally expanded cell lines). (D) Tabular overview of RT-qPCR Ct-value averages of *FGFR2* and *FGFR3* in HPK (N = 3–6 cultures from three different donors) and HaCaT keratinocytes (N = 3–6 cultures from three to four different passages). (E, F) RT-qPCR for (E) *FGFR1* or (F) *FGFR4* relative to *RPL27* using RNA from human primary fibroblasts (HPF) (N = 3 cultures from two different donors) or human hepatoma cells (HepG2) (N = 4 cultures), respectively, and from HPK (N = 2–6 cultures from three different donors) and the HaCaT cell line (N = 6 culturesfrom four different passages). (G) Representative FGFR2 or FGFR3 immunofluorescence (IF) staining of HPK, untreated or treated for 2 days with 1.5 mM Ca^2+^ and of low or high density HaCaT cells; counterstained with Hoechst (blue). Scale bars: 10 µm. Graphs show mean and standard deviation (SD). In (E) and (F), mean values for HPF and HepG2 were set to 1. ****P < 0.0001 (One-way ANOVA with Bonferroni’s multiple comparisons test (E, F)).

Analysis of bulk RNA-seq data from skin biopsies and HPK^19^ and from HaCaT keratinocytes (GSE267530) revealed very weak expression of *FGFR1* and *FGFR4*, but stronger expression of *FGFR2* and *FGFR3* in total skin and in both types of keratinocytes (Figure 1A–C). This was confirmed by RT-qPCR (Figure 1D–F). Expression of *FGFR1* and *FGFR4* was generally very low in keratinocytes in comparison to human primary fibroblasts (HPF) or HepG2 hepatoma cells, respectively (Figure 1E and F). Immunofluorescence (IF) staining revealed membrane and cytoplasmic localization of FGFR2 in HPK and in low-density HaCaT cells (Figure 1G). Upon addition of calcium, which results in differentiation of HPK,^20^ FGFR2 accumulated at the plasma membrane as previously described.^8^ A similar membrane accumulation was observed in HaCaT cells when they were grown to higher confluency (Figure 1G), which also induces differentiation.^21^ FGFR3 exhibited predominantly a cytoplasmic localization (Figure 1G).

The similar expression and localization of FGF receptors in HaCaT cells and HPK underscores the suitability of HaCaT cells as a model to study the function of FGF receptors in keratinocytes. Because of its strong expression and localization at the plasma membrane as well as the data from mouse studies, we focused our work on FGFR2.

### Generation of *FGFR2* knockout keratinocyte cell lines

**Figure 2.**
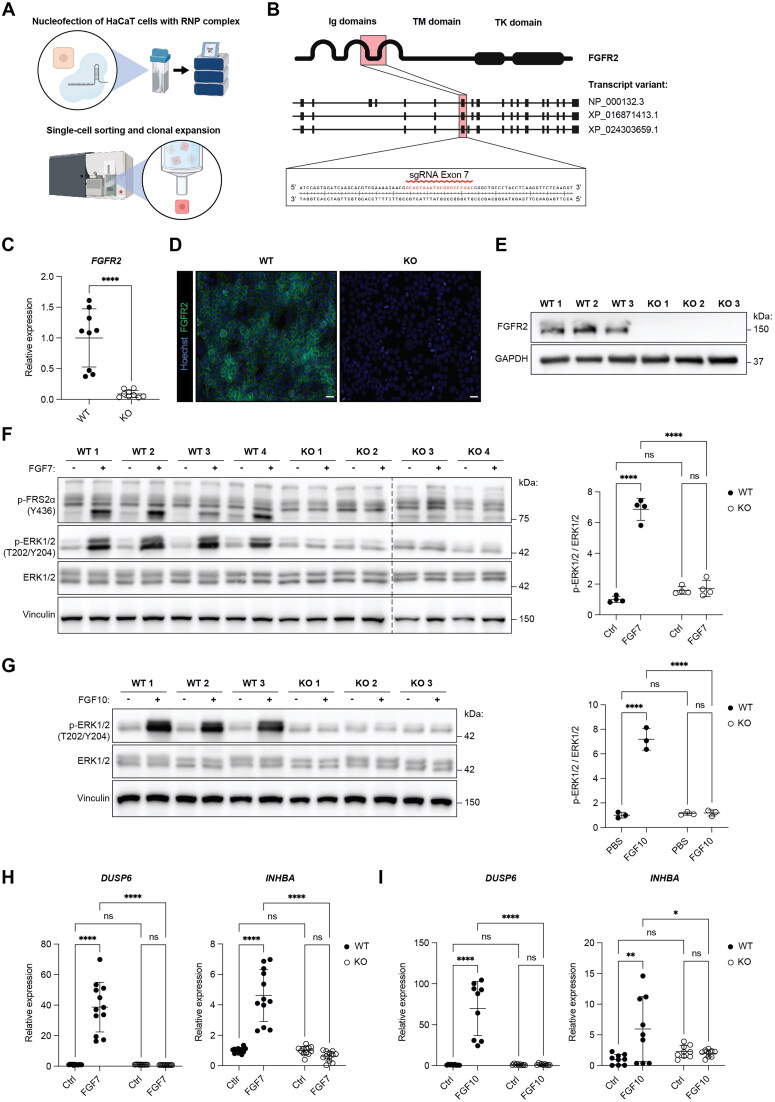
Generation of FGFR2-deficient HaCaT cell lines. (A) Schematic representation of the experimental procedure used to generate *FGFR2* KO HaCaT cell lines. (B) Schematic overview of the human genomic *FGFR2* sequence and the targeted exon 7, showing the sgRNA sequence targeting exon 7 and the corresponding domain of the protein. (C) RT-qPCR for *FGFR2* relative to *RPL27* using RNA from WT and *FGFR2* KO HaCaT cell lines (N = 3 cultures of three WT and three KO cell lines). (D) Representative IF staining of WT and *FGFR2* KO HaCaT cell lines for FGFR2 (green); counterstained with Hoechst (blue). Scale bar: 50 µm. (E) Western blot of lysates from WT and *FGFR2* KO HaCaT cell lines for FGFR2 and glyceraldehyde-3-phosphate-dehydrogenase (GAPDH; loading control) (N = 1 culture of three WT and three KO cell lines). (F) Western blot of lysates from serum-starved WT and *FGFR2* KO HaCaT cell lines, incubated for 10 min with 10 ng/ml FGF7 or vehicle. Membranes were probed with antibodies against p-FRS2α (Y436), p-ERK1/2 (T202/Y204), total ERK1/2 and vinculin (loading control). Densitometric quantification of p-ERK1/2 normalized to the intensity of total ERK1/2 is shown in the graph (N = 1 culture of four WT and four KO cell lines). (G) Western blot of lysates from serum-starved WT and *FGFR2* KO HaCaT cell lines, incubated for 10 min with 10 ng/ml FGF10 or vehicle. Membranes were probed with antibodies against p-ERK1/2 (T202/Y204), total ERK1/2 and vinculin. Densitometric quantification of p-ERK1/2 normalized to the intensity of total ERK1/2 is shown in the graph (N = 1 culture of three WT and three KO cell lines). (H, I) RT-qPCR for *DUSP6* and *INHBA* using RNA from serum-starved WT and *FGFR2* KO HaCaT cell lines, incubated for 6 h with 10 ng/mL (H) FGF7, (I) FGF10 or vehicle (N = 3 cultures of three to four WT and three to four KO cell lines). Graphs show mean and SD. Mean of control of WT cell lines was set to 1. Nonsignificant (ns), *P < 0.05, **P < 0.01, ****P < 0.0001 (Mann–Whitney U test (C), or 2-way ANOVA with Bonferroni’s multiple comparisons test (F-I)).

We next used CRISPR/Cas9 technology to generate FGFR2-deficient HaCaT keratinocytes (Figure 2A). After testing three single guide RNAs (sgRNAs) in pilot experiments, an efficient sgRNA was identified, which targets exon 7 of *FGFR2*. This is expected to result in the deletion of the transmembrane and the intracellular domains (Figure 2B). Following clonal expansion (Figure 2A), four wild-type (WT 1–4) and four FGFR2-deficient HaCaT cell lines (KO 1–4) were selected for further characterization. WT HaCaT cell lines consisted of the original cell population used for the generation of KO cell lines (WT 1) and clonally expanded WT clones (WT 2, 3, 4).

FGFR2 mRNA levels were strongly and significantly reduced in all tested KO cell lines, indicating that the *FGFR2* targeting almost completely abrogated gene expression and/or reduced the mRNA stability. Notably, expression of *FGFR2* varied between the different WT cell lines (Figure 2C). IF confirmed the predominant localization of FGFR2 at the cell membrane in confluent WT cells and its loss in the KO cell lines (Figure 2D), which was further supported by Western blot analysis (Figure 2E).

To examine if *FGFR2* signaling is indeed abolished, we treated HaCaT cells with FGF7, which exclusively activates FGFR2b.^5^ A 10 min incubation with FGF7 resulted in tyrosine phosphorylation of the downstream targets FGF receptor substrate (FRS)2α and ERK1/2 in all WT, but not in the KO cell lines (Figure 2F). This was also seen with FGF10 (Figure 2G), which primarily activates FGFR2b and to a lesser extent FGFR1b.^5^ Consistently, a 6 h treatment with FGF7 or FGF10 strongly induced the expression of the FGFR2 target genes *DUSP6* (encoding dual-specific phosphatase 6)^22^ and *INHBA* (encoding activin A)^23^ in WT, but not in KO HaCaT cells (Figure 2H and I).

### *FGFR2* KO HaCaT cells do not upregulate other FGF receptors

**Figure 3.**
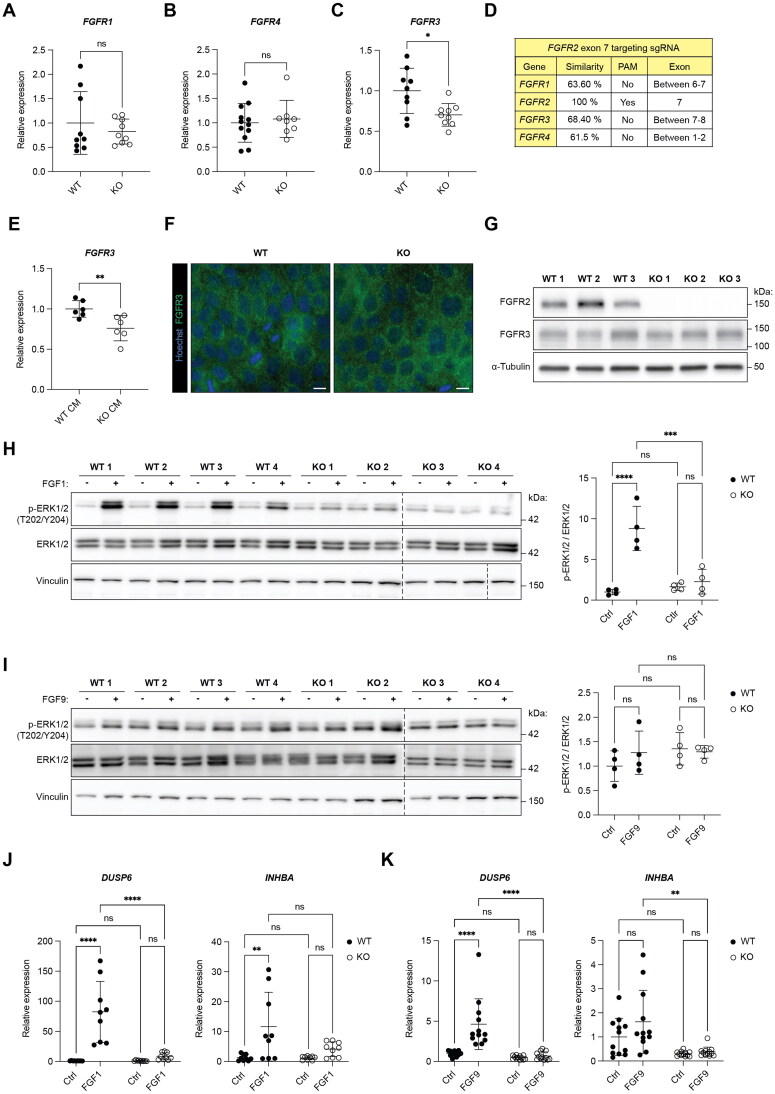
FGFR2 is the major functional FGF receptor in human keratinocytes. (A, B, C) RT-qPCR for (A) *FGFR1*, (B) *FGFR4* and (C) *FGFR3* relative to *RPL27* using RNA from WT and *FGFR2* KO HaCaT cell lines (N = 2–3 cultures of three to four WT and three KO cell lines). (D) Tabular overview of potential off-target sites for the sgRNA targeting exon 7 of *FGFR2* showing percentage of sequence homology with *FGFR1*, *FGFR2*, *FGFR3* and *FGFR4,* the presence or absence of a protospacer adjacent motif (PAM), and the genomic region of the homologous sequence motif. (E) RT-qPCR for *FGFR3* using RNA from wild-type HaCaT cells, incubated for 6 h with conditioned medium (CM) obtained from WT or *FGFR2* KO HaCaT cell lines (N = 3 cultures treated with CM of two WT or two KO cell lines). (F) Representative IF staining of WT and *FGFR2* KO HaCaT cell lines for FGFR3 (green); counterstained with Hoechst (blue). Scale bar: 10 µm. (G) Western blot analysis of lysates from WT and *FGFR2* KO HaCaT cell lines for FGFR2, FGFR3 and α-tubulin (loading control) (N = 1 culture of three WT and three KO cell lines). (H, I) Western blot analysis of lysates from serum-starved WT and *FGFR2* KO HaCaT cell lines, incubated for 10 min with 10 ng/mL (E) FGF1, (F) FGF9 or vehicle. Membranes were probed with antibodies against p-ERK1/2 (T202/Y204), total ERK1/2 and vinculin. Graph shows densitometric quantification of p-ERK1/2 normalized to the intensity of total ERK1/2 (N = 1 culture of four WT and four KO cell lines). (J, K) RT-qPCR for *DUSP6* and *INHBA* using RNA from serum-starved WT and *FGFR2* KO HaCaT cell lines, incubated for 6 h with 10 ng/mL (J) FGF1, (K) FGF9 or vehicle (N = 3 cultures of three to four WT and three to four KO cell lines). Graphs show mean and SD. Mean of control of WT cell lines was set to 1. Nonsignificant (ns), *P < 0.05, **P < 0.01, ***P < 0.001, ****P < 0.0001 (Mann–Whitney U test (A, B, C, E), or 2-way ANOVA with Bonferroni’s multiple comparisons test (H, I, J, K)).

We next investigated whether loss of FGFR2 leads to compensatory upregulation of other FGFRs. However, there was no difference in *FGFR1* or *FGFR4* expression between the WT and KO cell lines under baseline conditions (Figures 3A and B), and expression of *FGFR3* was even significantly downregulated (Figure 3C). We identified a 68.4% homology to intron sequences of *FGFR3*, but there was no protospacer adjacent motif (PAM) (Figure 3D), arguing against an off-target effect that affects *FGFR3*. A similar homology and lack of a PAM sequence were also observed for *FGFR1* and *FGFR4* (Figure 3D). Therefore, the loss of FGFR2 suppresses expression of *FGFR3*. This effect is most likely indirect, because treatment of WT HaCaT cells with conditioned medium (CM) of *FGFR2* KO cells, but not with CM of WT cells, also resulted in reduced *FGFR3* expression, demonstrating that FGFR2-deficient keratinocytes release factors, which are responsible for this effect (Figure 3E). However, there was no change in FGFR3 localization (Figure 3F) and no obvious reduction of FGFR3 protein levels in *FGFR2* KO compared to WT cells (Figure 3G).

To determine if activation of the remaining FGF receptors on HaCaT cells induces efficient intracellular signaling, we treated the cells with FGF1, a promiscuous ligand that binds to all FGFRs.^3^ ERK1/2 was strongly activated in all WT cells upon FGF1 treatment. However, the extent of FGF1-mediated ERK1/2 phosphorylation was much lower in two KO cell lines and even undetectable in the other two KO cell lines (Figure 3H). FGF9, which has a higher affinity for FGFR3 than for FGFR1,^3^^,^^5^ induced only a mild and nonsignificant increase in ERK1/2 phosphorylation (Figure 3I), which was lost in the *FGFR2* KO cell lines (Figure 3I). Consistent with the ERK1/2 activation, FGF1 induced the expression of *DUSP6* and *INHBA* in WT HaCaT cells, while *FGFR2* KO cells exhibited only a minor and nonsignificant increase in the expression of these genes (Figure 3J). FGF9 had a mild effect on *DUSP6* expression and no significant effect on *INHBA* expression in all WT cell lines. The FGF9-mediated increase in *DUSP6* expression was abrogated in all KO cell lines (Figure 3K). These findings identify FGFR2 as the primary FGF receptor in HaCaT keratinocytes, while signaling through the other FGF receptors is negligible.

### Loss of FGFR2 in keratinocytes does not affect EGFR signaling or the response to fetal bovine serum (FBS)

**Figure 4.**
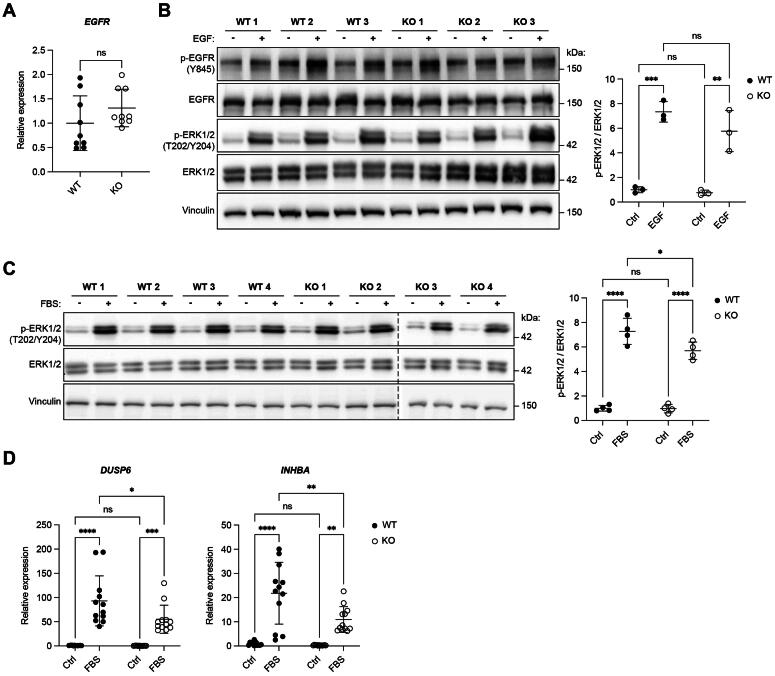
Loss of FGFR2 in HaCaT keratinocytes does not result in compensatory upregulation or activation of EGFR or activation of other growth factor receptors. (A) RT-qPCR for *EGFR* relative to *RPL27* using RNA from WT and *FGFR2* KO HaCaT cell lines (N = 3 cultures of three WT and three KO cell lines). (B) Western blot of lysates from serum-starved WT and *FGFR2* KO HaCaT cell lines, incubated for 10 min with 10 ng/mL EGF or vehicle. Membranes were probed with antibodies against p-EGFR (Y845 – a site that is phosphorylated by Src upon ligand stimulation^56^), total EGFR, p-ERK1/2 (T202/Y204), total ERK1/2 and vinculin. Graph shows densitometric quantification of p-ERK1/2 normalized to the intensity of total ERK1/2 (N = 1 culture of three WT and three KO cell lines). (C) Western blot of lysates from serum-starved WT and *FGFR2* KO HaCaT cell lines, incubated for 10 min with 10% FBS. Membranes were probed with antibodies against p-ERK1/2 (T202/Y204), total ERK1/2 and vinculin. Graph shows densitometric quantification of p-ERK1/2 normalized to the intensity of total ERK1/2 (N = 1 culture of four WT and four KO cell lines). (D) RT-qPCR for *DUSP6* and *INHBA* using RNA from serum-starved WT and *FGFR2* KO HaCaT cell lines, incubated for 6 h with 10% FBS (N = 3 cultures of three to four WT and three to four KO cell lines). Graphs show mean and SD. Mean of control of WT cell lines was set to 1. Nonsignificant (ns), *P < 0.05, **P < 0.01, ***P < 0.001, ****P < 0.0001 (Mann–Whitney U test (A), or 2-way ANOVA with Bonferroni’s multiple comparisons test (B-D)).

We next tested if the epidermal growth factor receptor (EGFR), which plays an important role in skin homeostasis^24^ and whose biological activities partially overlap with those of FGFRs,^25^ is hyperactivated in the KO cell lines as a compensatory mechanism. However, neither the expression of this receptor nor its EGF-induced phosphorylation at tyrosine 845, nor phosphorylation of the downstream target ERK1/2, differed between WT and *FGFR2* KO HaCaT cells (Figure 4A and B).

Treatment of HaCaT cells with FBS, which includes a wide variety of growth factors, induced strong phosphorylation of ERK1/2 in both WT and *FGFR2* KO cell lines (Figure 4C). The increase in ERK1/2 phosphorylation was slightly lower in the KO cells, possibly because of the reduced responsiveness to FGFs, which are also present in serum. Consistently, stimulation with FBS led to increased expression of *DUSP6* and *INHBA* in both WT and KO cell lines, although the upregulation was less pronounced in *FGFR2* KO cells (Figure 4D).

These results suggest that loss of FGFR2 does not induce a compensatory upregulation and activation of other receptors, including EGFR, which respond to growth factors present in serum.

### Knockout of *FGFR2* impairs FGF7-induced keratinocyte proliferation and migration

**Figure 5.**
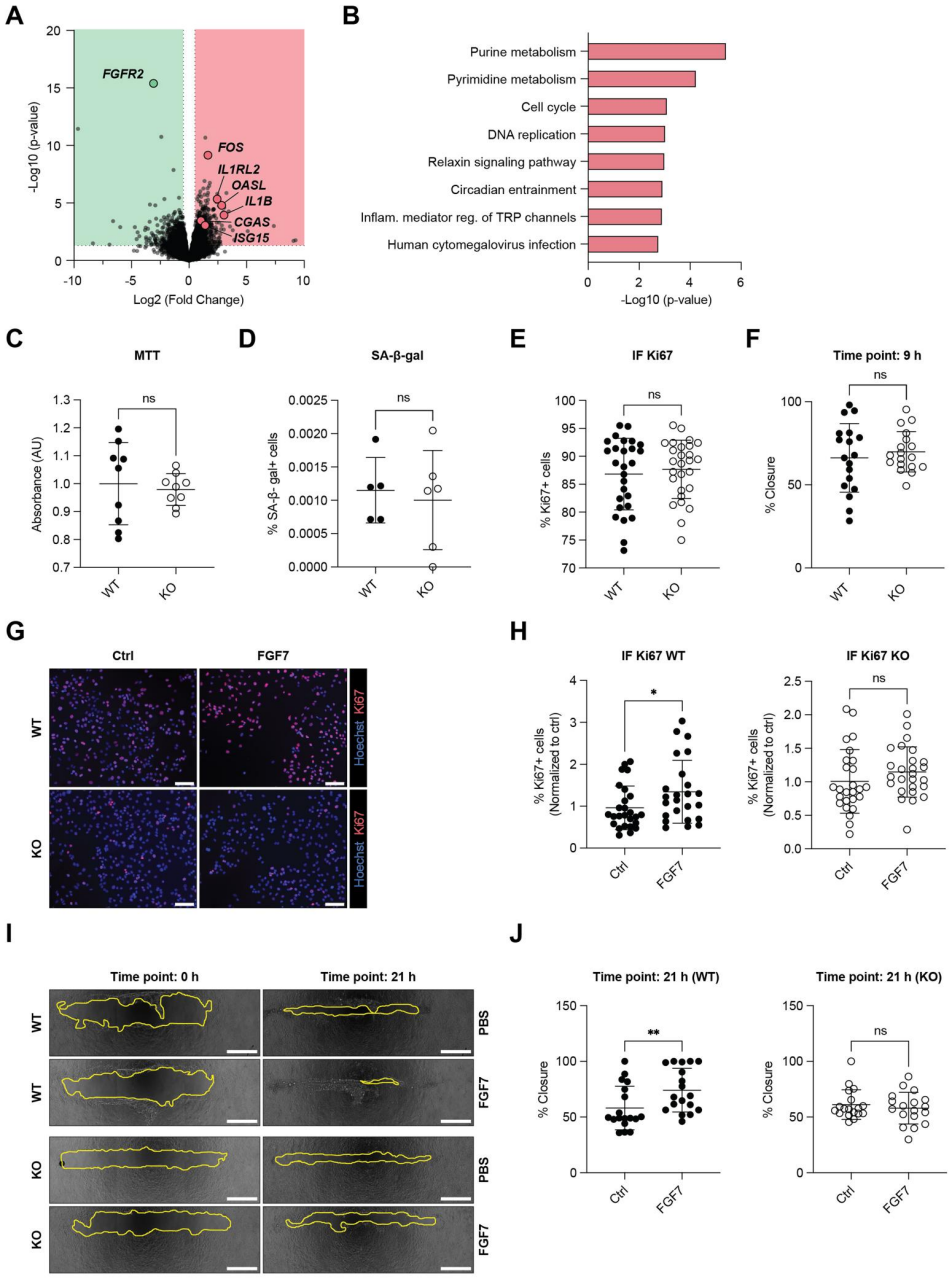
Loss of FGFR2 does not affect viability, proliferation or migration of HaCaT keratinocytes in full growth medium. (A) Volcano blot showing RNA-seq data from WT and *FGFR2* KO HaCaT cell lines (N = 3 cultures of three WT and three KO cell lines). Strongly regulated genes are indicated. (B) KEGG human pathway enrichment analysis based on the differentially expressed genes (*P* ≤ 0.05, FDR ≤ 0.05) in *FGFR2* KO HaCaT vs WT cells. (C) Cell viability assessed by MTT assay. Graph shows formazan absorbance after 30 min incubation with MTT (5 mg/mL) in WT and *FGFR2* KO HaCaT cell lines (N = 3 cultures of three to four WT and three to four KO cell lines). (D) Percentage of β-galactosidase^+^ (senescent) WT or *FGFR2* KO HaCaT cells (N = 2–3 cultures of two WT and two KO cell lines). (E) Percentage of cells with Ki67^+^ nuclei in WT or *FGFR2* KO HaCaT cells (N = 9 cultures of three WT and three KO cell lines). (F) Percentage of scratch closure of mitomycin C pre-treated WT and *FGFR2* KO HaCaT cell lines after 9 h (N = 6 cultures of three WT and three KO cell lines). (G) Representative Ki67 IF staining (red) of serum-starved WT and *FGFR2* KO HaCaT cell lines treated with FGF7 (10 ng/mL) or vehicle overnight; counterstained with Hoechst (blue). Scale bar: 100 µm. (H) Percentage of cells with Ki67^+^ nuclei in serum-starved WT and *FGFR2* KO HaCaT cell lines treated with FGF7 (10 ng/mL) or vehicle overnight (N = 8–9 cultures of three WT and three KO cell lines). (I) Representative images of scratch migration assay of WT and *FGFR2* KO HaCaT cell lines at 0 and 21 h. Scale bar: 500 µm. (J) Percentage of scratch closure of mitomycin C-pre-treated and serum-starved WT and *FGFR2* KO HaCaT cell lines treated for 21 h with FGF7 (10 ng/mL) or vehicle (N = 6 cultures of three WT and three KO cell lines). Graphs show mean and SD. In (C) and (H), mean values for control were set to 1. Nonsignificant (ns), *P < 0.05, **P < 0.01 (Mann–Whitney U test).

To identify differentially expressed genes (DEG) in *FGFR2* KO vs WT cells, we performed RNA sequencing (RNA-seq). Among the 241 differentially expressed genes (*P* ≤ 0.05 and FDR ≤ 0.05), 56 displayed decreased expression (log2FC ≤ -0.5), with *FGFR2* being strongly downregulated. One hundred and eighty-four genes exhibited significantly increased expression (log2FC ≥ 0.5), including *FOS*, *IL1RL2*, *OASL*, *IL1B*, *CGAS*, and *ISG15* (Figure 5A). Pathway enrichment analysis of the DEG revealed enrichment of purine and pyrimidine metabolism, cell cycle, and DNA replication, which point to alterations in cell proliferation and potentially survival (Figure 5B). However, there was no difference in the viability, the rate of proliferation and the number of senescent (β-galactosidase positive) KO cells when they were cultured in serum-containing medium (Figure 5C–E). In addition, the migratory capabilities of the WT and *FGFR2* KO cell lines were comparable in a scratch assay (Figure 5F). As expected, however, FGF7 induced a mild, but significant increase in proliferation and migration in serum-free medium, which was abrogated in the *FGFR2* KO cells (Figure 5G–J).

### Knockout of *FGFR2* induces expression of ISGs

**Figure 6.**
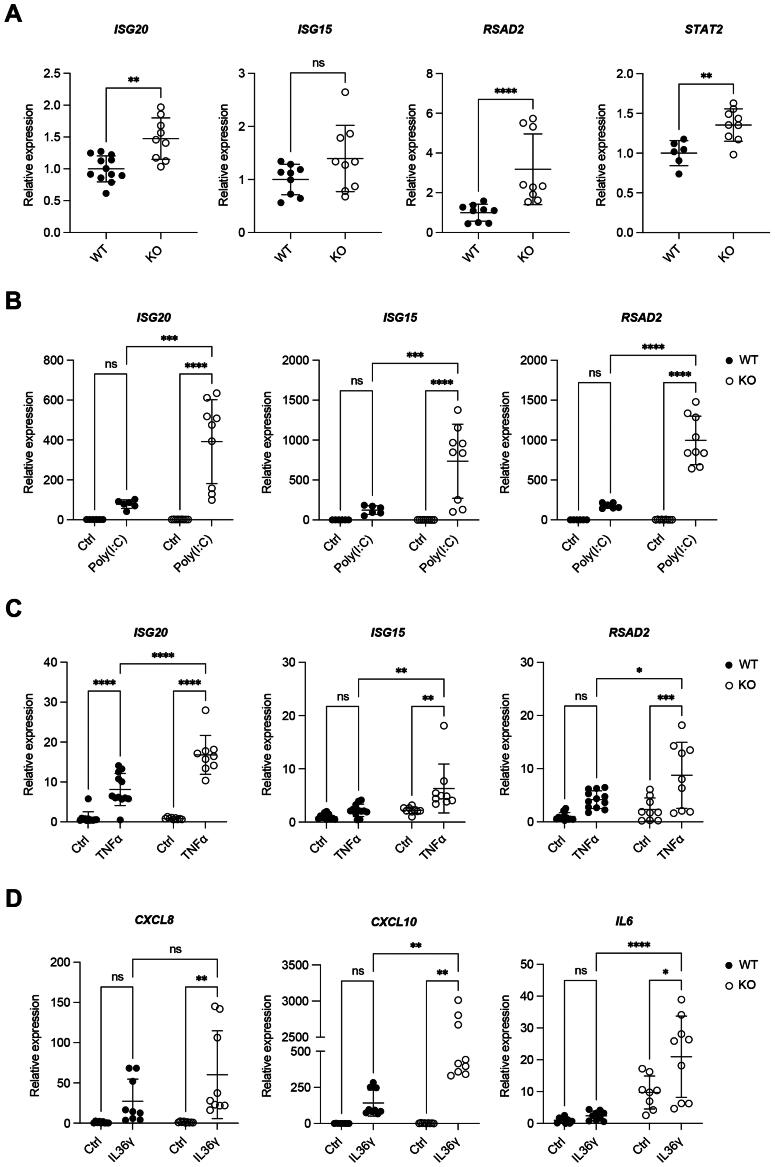
Loss of FGFR2 in HaCaT keratinocytes promotes the expression of interferon-stimulated genes (ISGs) and of pro-inflammatory chemokines. (A) RT-qPCR for *ISG20*, *ISG15*, *RSAD2* and *STAT2* relative to *RPL27* using RNA from WT and *FGFR2* KO HaCaT cell lines (N = 3 cultures of two to three WT and three KO cell lines). (B, C) RT-qPCR for *ISG20*, *ISG15* and *RSAD2* using RNA from WT and *FGFR2* KO HaCaT cell lines, incubated for 6 h with (B) 1 µg/mL poly(I:C), (C) 20 ng/mL TNFα or vehicle (N = 3 cultures of two to four WT and three to four KO cell lines). (D) RT-qPCR for *CXCL8*, *CXCL10* and *IL6* using RNA from WT and *FGFR2* KO HaCaT cell lines, incubated for 6 h with 100 ng/mL IL-36γ or vehicle (N = 3 cultures of three WT and three KO cell lines). Graphs show mean and SD. Control of WT cell lines was set to 1. Nonsignificant (ns), *P < 0.05, **P < 0.01, ***P < 0.001, ****P < 0.0001 (Mann–Whitney U test (A), two-way ANOVA with Bonferroni’s multiple comparisons test (B-D)).

Because of the observed pathway enrichment of “human cytomegalovirus infection” in the RNA-Seq analysis of *FGFR2* KO HaCaT cells (Figure 5B) and the previously demonstrated suppression of the expression of a battery of antiviral and/or pro-inflammatory ISGs in keratinocytes by FGF7 and FGF10,^18^ we determined if long-term genetic loss of *FGFR2* in human keratinocytes has the opposite effect. Indeed, we observed a significantly increased expression of *ISG20*, *RSAD2* (encoding radical S-adenosyl methionine domain containing 2) and *STAT2* (encoding signal transducer and activator of transcription 2) and a non-significant increase in *ISG15* expression in *FGFR2* KO compared to WT cells, even in the absence of a pro-inflammatory stimulus (Figure 6A).

Treatment of the different WT and KO cell lines with polyinosinic:polycytidylic acid (poly(I:C)), a synthetic analogue of dsRNA, which activates toll-like receptor 3 (TLR3), resulted in the expected increase in *ISG20*, *ISG15* and *RSAD2* expression in all cell lines. Importantly, however, the activation of these genes in response to poly(I:C), and also to tumor necrosis factor α (TNFα), was strongly increased in *FGFR2* KO HaCaT cells (Figure 6B and C). These results demonstrate a key role of FGFR2 as a suppressor of ISG expression, in particular in response to a pro-inflammatory stimulus.

An additional gene that was upregulated in *FGFR2* KO keratinocytes (Figure 5A) encodes the receptor for interleukin (IL)-36 cytokines (IL-36R, also known as IL1RL2). This finding is intriguing, because of the important role of IL-36 family members in the pathogenesis of AD and other inflammatory skin diseases,^26^^,^^27^ and the heightened IL-36 response in the transition from non-lesional to acute to chronic AD skin.^26^ Therefore, we investigated whether the KO cells are more susceptible to activation of the IL-36R. Treatment of WT cells with IL-36γ resulted in a mild, but nonsignificant increase in the expression of *CXCL8*, *CXCL10* and *IL6* (Figure 6D), which are classical target genes of IL-36R signaling.^28^ Notably, activation of these genes by IL-36γ was significantly stronger in the FGFR2-deficient cells (Figure 6D). This finding demonstrates that FGFR2 controls the response to pro-inflammatory stimuli beyond ISG expression, e.g., by increasing the responsiveness to IL-36 cytokines.

### Expression of *FGFR2* is suppressed by pro-inflammatory mediators and downregulated in AD epidermis

**Figure 7.**
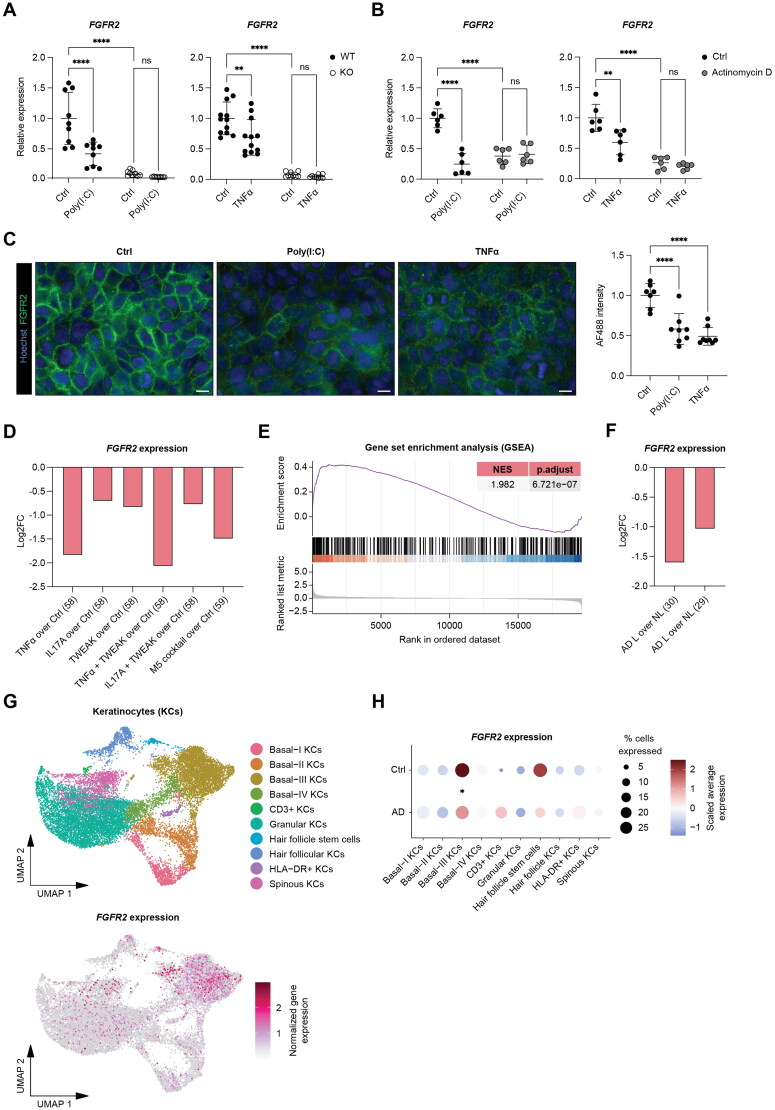
Suppression of *FGFR2* expression by pro-inflammatory stimuli. (A) RT-qPCR for *FGFR2* relative to *RPL27* using RNA from WT and *FGFR2* KO HaCaT cell lines, incubated for 6 h with 1 µg/mL poly(I:C), 20 ng/mL TNFα or vehicle (N = 3 cultures of three to four WT and three KO cell lines). (B) RT-qPCR for *FGFR2* using RNA from WT and *FGFR2* KO HaCaT cell lines, pre-treated for 1 h with 1 µg/mL actinomycin D or vehicle and incubated for 6 h with 1 µg/mL poly(I:C) or 20 ng/mL TNFα or vehicle (N = 3 cultures of two independent experiments). (C) Representative immunofluorescence images of HaCaT cells treated for 16 h with 1 µg/mL poly(I:C) or 20 ng/mL TNFα or vehicle using antibodies against FGFR2 (green) and counterstained with Hoechst (blue). Scale bars: 10 µm. Graph shows quantification of FGFR2 mean IF staining intensity (N = 2–3 cultures of three independent experiments). (D) *FGFR2* log2FC expression changes in HPK upon treatment with 100 ng/mL recombinant TNF-like weak inducer of apoptosis (TWEAK), 100 ng/mL human recombinant IL-17A, 10 ng/mL human recombinant TNFα,or a combination of TWEAK with IL-17A or TNFα compared to respective control^57^, or in N/TERT immortalized keratinocytes upon treatment with M5 inflammatory cytokine cocktail containing IL-17A, IL-1α, oncostatin M, TNFα and IL-22 at 10 ng/mL each compared to control.^58^ (E) GSEA plot showing significant enrichment of genes upregulated in the epidermis of lesional AD skin^29^ in ranked gene sets obtained from the epidermis of mice lacking Fgfr1 and Fgfr2 in keratinocytes.^18^ NES: Normalized enrichment score; p.adjust: adjusted *P* value. (F) *FGFR2* log2FC expression changes in lesional vs non-lesional skin from AD patients (total skin^30^ and laser-dissected epidermis^29^) (G) UMAP representation of skin keratinocyte subsets (defined by keratin expression)^31^ colored by annotated keratinocyte subtypes (top) and expression of *FGFR2* (bottom). (H) Dot plot showing *FGFR2* expression by annotated cluster and condition, highlighting keratin-positive subsets with differential *FGFR2* expression between AD and control. Graphs show mean and SD. In (A-C) mean values for control were set to 1. Nonsignificant (ns), *P < 0.05, **P < 0.01, ****P < 0.0001 (2-way ANOVA with Bonferroni’s multiple comparisons test (A, B), Wilcoxon rank sum test (H)).

Given the increased ISG expression in keratinocytes in the absence of FGFR2, we investigated if inflammatory mediators affect the expression of *FGFR2*. Intriguingly, we found a significant suppression of *FGFR2* expression in HaCaT keratinocytes following treatment with poly(I:C) or TNFα (Figure 7A). This was abrogated by pre-incubation of the cells with actinomycin D, which inhibits transcription (Figure 7B). A 16 h exposure to poly(I:C) or TNFα also reduced the levels of FGFR2 protein (Figure 7C). Suppression of *FGFR2* expression also occurred in different types of human keratinocytes in response to other pro-inflammatory stimuli, as shown by analysis of published transcriptome data from cultured HPK or immortalized N/TERT keratinocytes, which had been treated with interleukin (IL)-17A, TNF-like weak inducer of apoptosis (TWEAK), TNFα, or a combination of these factors, or with an inflammatory cytokine cocktail named "M5" (Figure 7D). These data demonstrate the conservation of this mechanism in primary and different types of immortalized human keratinocytes.

The downregulation of FGFR2 expression by pro-inflammatory stimuli pointed to a potential role of this receptor in the pathogenesis of inflammatory skin disease. Consistent with this assumption, a comparison of differentially expressed genes in mice lacking Fgfr1 and Fgfr2 in keratinocytes^18^ with microarray data from laser-capture isolated epidermis of lesional AD and control skin^29^ via gene set enrichment analysis (GSEA) showed a significant enrichment of the upregulated genes (Figure 7E). In addition, analysis of published transcriptome data revealed strong downregulation of *FGFR2* in lesional skin of AD patients^30^ and even in laser capture isolated epidermis from these patients (Figure 7F).^29^ Supporting these observations, single cell (sc)RNA-Seq data from lesional skin of AD patients showed reduced FGFR2 mRNA levels, particularly in a subpopulation of basal keratinocytes (Basal-III KCs) (Figure 7G and H).^31^^,^^32^ An exception was a very small population of cells (1–2% of all keratin-positive cells), which expressed keratins and also the T cell markers CD3. The origin of these cells is as yet unknown. They may represent a new type of intraepidermal T cells, which express keratinocyte markers. Expression of *FGFR2* was mildly, although nonsignificantly increased in this population in AD vs healthy skin.

Taken together, these results identify FGFR2 as a novel player in the pathogenesis of this common skin disease and an FGFR-mediated regulatory mechanism that controls skin inflammation via keratinocytes. Furthermore, they strengthen the important role of keratinocyte signaling in AD.^33^

## Discussion

We discovered an unexpected role of FGFR2 signaling in keratinocytes as a suppressor of skin inflammation. The additional loss of Fgfr1 in keratinocytes aggravated the phenotype of Fgfr2-deficient mice,^15^ most likely because of the overlapping ligand binding specificities of Fgfr1b and Fgfr2b.^5^ Thus, the low levels of Fgfr1 expressed in mouse keratinocytes become relevant in the absence of Fgfr2. It is possible that additional loss of FGFR1 in FGFR2-deficient HaCaT cells further aggravates their phenotype, although the very low responsiveness of *FGFR2* KO HaCaT cells to FGF1 suggests a minor role of FGFR1 in human keratinocytes.

The knockout of *FGFR*2 in HaCaT keratinocytes did not induce obvious compensatory responses via other FGF receptors or other growth factor receptors, and even suppressed the expression of *FGFR3*, which is consistent with the *Fgfr3* downregulation in mice lacking Fgfr1 and Fgfr2 in keratinocytes.^16^ Even in wild-type cells, activation of FGFR3 in HaCaT cells by FGF9 initiated only a very mild response, in spite of its high expression. These findings are in line with the normal growth of HPK with shRNA-mediated knock-down of FGFR3.^34^ Because strong FGFR3 staining was previously detected in suprabasal, differentiated keratinocytes of human skin,^35^ the responsiveness of keratinocytes to FGFR3 ligands may increase during the process of differentiation.

Characterization of the FGFR2-deficient human keratinocytes showed the expected loss of FGF7-induced keratinocyte proliferation and migration. However, no differences in these parameters were observed in serum-containing medium. Although compensatory upregulation and activation of other receptors and signaling pathways, including EGFR signaling, was not observed, it is likely that other growth factors present in the serum-containing medium offset the absence of FGFR2, enabling normal keratinocyte growth and migration in full medium. This is due to the redundant functions of these factors in supporting keratinocyte survival, proliferation and migration.

Transcriptomic analysis of the *FGFR2* KO HaCaT cells showed upregulation of several ISGs under normal culture conditions and in particular in response to inflammatory stimuli. ISGs are a large gene family that encode proteins with important functions in pathogen defense, immune regulation and inflammation.^36^ Their increased expression in FGFR2-deficient keratinocytes points to a key role of this receptor in the control of these processes. Indeed, we previously showed that FGFR kinase inhibitors suppress the infection of keratinocytes with herpes simplex virus I, while FGF7 or FGF10 had the opposite effect.^18^ The data obtained in the present study show that the loss of FGFR2 in human keratinocytes promotes the responsiveness of keratinocytes to pro-inflammatory stimuli. This is consistent with the spontaneous development of an AD-like phenotype in mice lacking Fgfr1 and Fgfr2 in keratinocytes^37^^,^^38^ and the upregulation of genes in the epidermis of these mice, which are also expressed at higher levels in the epidermis of lesional skin from AD patients according to GSEA (this study). The AD-like phenotype in Fgfr1/Fgfr2-deficient mice has been attributed to the downregulation of tight junction proteins, resulting in a defect of the epidermal barrier.^15^ An impaired barrier is well known to cause inflammatory responses by allowing invasion of irritants, allergens and pathogens, as seen for example in AD.^39^ In addition, it results in enhanced trans-epidermal water loss and dry skin, which may further promote skin inflammation.^39^ Indeed, exposure of Fgfr1/Fgfr2-deficient mice to high environmental humidity partially rescued the skin phenotype, although the enhanced expression of pro-inflammatory cytokines was not completely suppressed.^38^ Our new data suggest that inflammation in AD skin also involves FGFR2 downregulation, resulting in enhanced expression of pro-inflammatory genes in keratinocytes. Consistent with this assumption, expression of ISGs is upregulated in the epidermis of mice lacking Fgfr1 and Fgfr2 in keratinocytes^18^ and also in lesional skin of AD patients.^29^^,^^40^ In addition, we found a higher expression of the IL-36R in FGFR2-deficient HaCaT cells and increased responsiveness of these cells to IL-36γ. This finding provides a possible explanation for the increased IL-36 response that was observed during the transition from non-lesional to acute and chronic AD.^26^

While our study discovered an important role of FGFR2-mediated signaling in inflammation, it has certain limitations. One limitation is the use of immortalized HaCaT keratinocytes. This was necessary because HPK cannot be clonally expanded. However, we showed that HaCaT cells have a similar FGFR expression pattern and FGF7 responsiveness as HPK, making them a good model to study this process. Nevertheless, they could not be used to study the effect of FGFR2 deficiency on keratinocyte differentiation in this experimental system, as clonally expanded HaCaT cells consistently lost their potential for terminal differentiation in 3D skin cultures. We did not further address this question, because the role of FGFR2 in keratinocyte differentiation was previously characterized. In these studies, siRNA-mediated knock-down of FGFR2 modulated early and late differentiation of keratinocytes in 2D cultures of HPK.^41^^,^^42^ Another limitation is the use of 2D cultures to study the effect of FGFR deficiency and the regulation of FGFR2 expression. In the future, it will be interesting to determine if the results obtained in this study can be verified in 3D organotypic cultures with fibroblasts. Finally, the *in vivo* data on FGFR downregulation in AD are based on scRNA-seq data, and it will be important to verify these results at the protein level. This will require the optimization of the FGFR2 staining procedure for tissue sections.

In spite of these limitations, our results identify a previously unknown role of FGFR2 in keratinocytes in the control of skin inflammation. FGF7 has also well documented cytoprotective effects on epithelial cells of different organs.^9^^,^^11^ This activity has been thought to result from its effect on the detoxification of reactive oxygen species and its direct anti-apoptotic activity.^13^ Our new findings strongly suggest that the anti-inflammatory function of FGF7 further contributes to its cytoprotective effect *in vivo*. Of particular relevance in this context is the unexpected downregulation of *FGFR2* expression in response to different inflammatory stimuli that we discovered in this study. This is of likely relevance *in vivo* as suggested by the downregulation of *FGFR2* expression in keratinocytes of lesional skin of AD patients. Our functional *in vitro* studies demonstrate that FGFR2 downregulation further promotes inflammation, thereby initiating a vicious cycle. Of note, a strong reduction of *FGFR2* expression was also observed in the inflamed gut of patients with inflammatory bowel disease.^43^ Therefore, the results obtained in the present study are of likely relevance for inflammatory processes in general. They pave the way for the development of strategies to promote the expression and activity of FGFR2 in epithelial cells for the prevention or treatment of excessive and prolonged inflammation.

## Materials and Methods

### Cell culture

Low-passage HaCaT keratinocytes^17^ and HepG2 human hepatoma cells (Sigma-Aldrich, St Louis, MO) were cultured in high-glucose Dulbecco’s Modified Eagle Medium (DMEM, Thermo Fisher Scientific, Waltham, MA) supplemented with 10% FBS (Life Technologies, Carlsbad, CA) at 37 °C and 5% CO_2_. Regular mycoplasma contamination screening was performed using the PCR Mycoplasma Test Kit I/C (PromoCell, Heidelberg, Germany), confirming the absence of contamination in all cell lines.

HPK and HPF were isolated from the foreskin of healthy donors without skin disease, as described previously.^44^ Foreskin samples were obtained anonymously from healthy boys with parental written consent, as part of the University of Zurich biobank project. The collection and use of samples were approved by the local and cantonal Research Ethics Committees, adhering to the Declaration of Helsinki Principles. HPK were cultured in Keratinocyte Serum-Free Medium (K-SFM) with EGF and bovine pituitary extract (BPE, all from Thermo Fisher Scientific) under standard conditions at 37 °C with 5% CO_2_, and culture medium was replaced 2–3 times per week. HPF were cultured in DMEM/10% FBS.

### Treatment of cells for functional experiments

Cells were seeded at a concentration of 350,000 cells/well into 12-well plates for RNA analysis or 700,000 cells/well into 6-well plates for protein analysis. After reaching confluency, they were either starved overnight (o/n) in DMEM without FBS or treated directly without starvation, as indicated for the respective experiments. Cells were incubated with the respective growth factors or inflammatory mediators for 6 h or o/n for RNA analysis, and for 10 min for protein analysis. The following proteins/compounds were used: Human FGF7, FGF1 or FGF9 (all 10 ng/mL, Peprotech, Cranbury, NJ), EGF (10 ng/mL; Sigma-Aldrich), poly(I:C) (1 μg/mL, InvivoGen, San Diego, CA), TNFα (20 ng/ml, Peprotech) or IL-36γ (100 ng/mL, Peprotech).

### Cell migration and proliferation assays

To assess the migratory capability, cells were grown to confluency in 96-well plates and starved o/n if necessary. The following day, they were washed once with phosphate buffered saline (PBS) and treated with mitomycin C (2 ng/mL; Sigma-Aldrich) in starvation medium or complete medium for 2 h. Scratches were generated using the 96 Floating E-Clip style Pin Multi-Blot Replicator and the alignment jig (both from V&P Scientific, San Diego, CA). Cells were washed once with PBS and treated with FGF7 or vehicle (PBS) in starvation medium or complete medium. Cells were imaged immediately (0 h) and at 3 h, 6 h, 9 h, 21 h, 24 h and 27 h after scratching. Image acquisition was performed with an Axio Observer Z1 inverted phase contrast fluorescence microscope equipped with A-Plan 10x/0,25 Ph1 objective, using the ZEN pro software (all from Carl Zeiss Inc., Oberkochen, Germany). Images were analyzed using the plugin “Wound healing size tool” in ImageJ/FIJI (National Institutes of Health, Bethesda, MD).

To assess proliferation, cells were grown to confluency in 24-well plates and starved o/n if necessary. The starved cells were washed once with PBS and treated o/n with PBS or FGF7 in starvation medium, or remained in complete medium. Fixation and immunofluorescence staining for Ki67 were performed as described below.

### CRISPR/Cas9-mediated knockout of *FGFR2*

sgRNAs targeting exon 7 were designed using CHOPCHOP.^45^ The following sgRNA, which includes a PAM sequence, was chosen for targeted knockout: 5′-GCAGTAAATACGGGCCCGACGGG-3′ (Thermo Fisher Scientific). For excision, Cas9 from *Streptococcus pyogenes* was utilized (Thermo Fisher), which recognizes the PAM-sequence 5′-NGG-3′. RNP complex formation, nucleofection and electroporation were performed as described previously.^46^ Nucleofection was performed using the P3 primary cell nucleofector kit S (Lonza AG, Basel, Switzerland). Electroporation was performed using the 4D-Nucleofector^®^ X Unit with the pulse code DS-138 (Lonza).

Knockout and control cell populations were expanded for a maximum timespan of 10 days and single-cell-sorted to allow for clonal expansion. Cells were trypsinized, resuspended in 1 mL sterile FACS-buffer (PBS; 1 mM EDTA; 25 mM HEPES, 7.0 pH; 1% BSA) and stained with SYTOX^™^ Green Dead Cell Stain (Thermo Fisher Scientific). Single cell sorting was performed using the BD FACSAria III sorter (BD Biosciences, Franklin Lakes, NJ). Afterward, single cells were expanded and screened for knockout of *FGFR2*.

### RNA isolation and RT-qPCR

RNA extraction was performed using the Mini Total Tissue RNA kit (IBI Scientific, Dubuque, IO), including a DNase digestion step, and the RNA was dissolved in 30 μL DEPC-H_2_O. One μg RNA was reverse transcribed into cDNA using the iScript cDNA synthesis kit (Bio-Rad, Hercules, CA), according to the manufacturer’s protocol.

For amplification, the cDNA was diluted 1:10, and 5 μL cDNA were mixed with 5.5 μL LightCycler SYBR green (Roche, Rotkreuz, Switzerland) and 0.5 μL primer mix (10 μM). qPCR was performed in 384-well plates, and all samples were run in technical duplicates. Relative expression levels were calculated using the 2^-ΔΔCt^ method,^47^ and gene expression was normalized to the expression of the housekeeping gene *RPL27*. Primer sequences are listed in Table 1.

**Table 1.** Primer sequences used for RT-qPCR.

Gene	Forward primer	Reverse primer
*FGFR1*	CCC GTA GCT CCA TAT TGG ACA	TTT GCC ATT TTT CAA CCA GCG
*FGFR2*	AGC TGG GGT CGT TTC ATC TG	TTG GTT GGT GGC TCT TCT GG
*FGFR3*	AGA TCG CAG ACT TCG GGC T	ATC CAC TTC ACG GGC AGC
*FGFR4*	GCA CTG GAG TCT CGT GAT GG	CCA CAG CGT TCT CTA CCA GG
*CXCL8*	CTG GCC GTG GCT CTC TTG	TTA GCA CTC CTT GGC AAA ACT G
*CXCL10*	CCA CGT GTT GAG ATC ATT GC	TGC ATC GAT TTT GCT CCC CT
*DUSP6*	GTT CTA CCT GGA AGG TGG CT	AGT CCG TTG CAC TAT TGG GG
*IL6*	GAT TCA ATG AGG AGA CTT GCC	TGT TCT GGA GGT ACT CTA GGT
*INHBA*	GGA GAA CGG GTA TGT GGA GA	ACA GGT CAC TGC CTT CCT TG
*ISG15*	CTT TGC CAG TAC AGG AGC T	GAC ACC TGG AAT TCG TTG C
*ISG20*	CTC GTT GCA GCC TCG TGA A	CGG GTT CTG TAA TCG GTG ATC TC
*RSAD2*	CCA GTG CAA CTA CAA ATG CGG C	CGG TCT TGA AGA AAT GGC TCT CC
*STAT2*	GGA TCC TAC CCA GTT GGC TG	GAG GGT GTC TTC CCT TTG GC
*RPL27*	TCA CCT AAT GCC CAC AAG GTA	CCA CTT GTT CTT GCC TGT CTT

### Preparation of protein lysates and Western blot analysis

Cells were washed twice with ice-cold PBS and lysed with 200 μL NP40 lysis buffer (1% NP40, 150 mM NaCl, 50 mM Tris, pH 7.8), containing PhosSTOP phosphatase inhibitor (1 tablet per 10 mL) and complete, EDTA-free protease inhibitor cocktail (1 tablet per 10 mL, both from Roche) for each well of a 6-well plate. Lysates were scraped off using a cell scraper, and the lysate was placed on a rotating platform for 30 min at 4 °C. Afterward, samples were centrifuged at 12,000 rpm for 15 min at 4 °C, and the supernatant was used for Western blot analysis. Protein concentration was determined using the Pierce Bicinchoninic Acid (BCA) Protein Assay (Thermo Fisher Scientific), and absorbance was measured at 560 nm using the GloMax^®^ Discover plate reader (Promega). Protein concentrations were calculated based on the standard protein concentration curve.

Proteins were separated by SDS-PAGE and transferred onto Protran^™^ 0.2 μm nitrocellulose membranes (Amersham, Amersham, UK). After washing with TBS-T (25 mM Tris base, 137 mM NaCl, 2.7 mM KCl, 0.1% Tween 20, pH 8.0), unspecific binding sites on the membranes were blocked with 5% bovine serum albumin (BSA; PAN Biotech, Aidenbach, Germany) in TBS-T for 1 h at room temperature (RT), following incubation with the primary antibody diluted in TBS-T containing 5% BSA o/n at 4 °C. The following day, the membranes were washed three times for 10 min each with TBS-T and incubated with the secondary antibody diluted in TBS-T containing 5% BSA for 1 h at RT. They were then washed three times for 10 min each with TBS-T, and the signal was developed using the Western Bright ECL kit (Advansta, San Jose, CA) and visualized with the FUSION SOLO 6S Western blot imaging system (Vilber Lourmat, Marne-La-Vallé, France). Primary and secondary antibodies are listed in Table 2. Band intensities were quantified using ImageJ/FIJI (NIH) and normalized to a housekeeping protein.

**Table 2.** Primary and secondary antibodies used for Western blot.

Antibody	Ordering number and supplier	Dilution
Primary antibodies		
Rabbit anti-phospho-FRS2α (Tyr436)	#3861, Cell Signaling Technology, Danvers, MA	1:500
Rabbit anti-ERK1/2	#102, Cell Signaling Technology	1:1000
Rabbit anti-phospho-ERK1/2	#9101, Cell Signaling Technology	1:1000
Mouse anti-vinculin	#v-4505, Sigma-Aldrich	1:500
Mouse anti-GAPDH	#5G4, HyTest, Turku, Finland	1:1000
Rabbit anti-FGFR2 (D4L2V)	#23328, Cell Signaling Technology	1:1000
Rabbit anti-FGFR3 (C51F2)	#4574, Cell Signaling Technology	1:1000
Mouse anti-EGFR	#sc-373746, Santa Cruz, Santa Cruz, CA	1:100
Rabbit anti-phospho-EGFR	#sc-2231, Cell Signaling Technology	1:1000
Mouse anti-α-tubulin	#T5168, Sigma-Aldrich	1:5000
Secondary antibodies		
Goat anti-rabbit IgG HRP conjugate	#W4018, Promega, Madison, WI	1:5000
Goat anti-mouse IgG HRP conjugate	#W402B, Promega	1:5000

### Immunofluorescence staining

Cells were seeded on glass coverslips in 24-well plates and washed three times with PBS before fixation with 4% paraformaldehyde for 15 min at RT. For FGFR2 staining, samples were then incubated for 10 min in cold 100% methanol at –20 °C. Permeabilization and blocking (Perblock buffer: PBS, 1% BSA and 0.3% Triton X-100) was performed for 1 h at RT. For Ki67 staining, permeabilization was performed in 0.2% Triton-X 100 in PBS for 10 min at RT, followed by blocking in 3% BSA and 0.025% NP-40 in PBS for 1 h at RT. Primary antibodies (Table 3) diluted in Perblock buffer (except for Ki67, which was diluted in 1% BSA in PBS) were diluted as shown below, and samples were incubated overnight at 4 °C in a humidified chamber. The next day, the secondary antibody Table 3 was diluted in Perblock buffer (except for goat anti-rabbit Cy3, which was diluted in 1% BSA in PBS), and samples were incubated for 1 h at RT. Hoechst (Sigma-Aldrich) staining was performed by diluting the stock 1:1000 in PBS and incubating for 5 min at RT. Samples were washed three times with PBS after each step. Mowiol (Sigma-Aldrich) was used to mount coverslips on microscopy slides, which were then air-dried in the dark before imaging with a Zeiss Imager.A1 microscope equipped with a Plan-Apochromat objective (63×/1.4 NA Oil) and an Axiocam MRm camera, using ZEN Blue software. Ki67 images were acquired with a Zeiss Axiovert Observer.Z1, equipped with an Axiocam 506 mono (all from Carl Zeiss Inc.). Quantification of the FGFR2 mean signal intensities was conducted using the open-source software QuPath,^48^ and images acquired using a ZEISS Axioscan 7 Microscope Slide Scanner (20×/0.8 NA).

**Table 3.** Primary and secondary antibodies used for immunofluorescence staining.

Antibody	Supplier	Dilution
Primary antibodies		
Rabbit anti-FGFR1	#ab10646, Abcam, Cambridge, UK	1:200
Rabbit anti-FGFR2 (D4L2V)	#23328, Cell Signaling Technology	1:250
Rabbit anti-FGFR3 (C51F2)	#4574, Cell Signaling Technology	1:200
Rabbit anti-Ki67	ab15580, Abcam	1:500
Secondary antibodies		
Donkey anti-rabbit IgG Alexa 488	#71-547-003, Jackson ImmunoResearch, West Grove, PA	1:400
Goat anti-rabbit-Cy3	#111-165-003, Jackson ImmunoResearch	1:200

### β-galactosidase staining

Cells were washed twice for 30 s with PBS and fixed in PBS containing 2% formaldehyde and 0.2% glutaraldehyde for 5 min at RT. After three washing steps with PBS for 30 s each, they were incubated in β-galactosidase staining solution (40 mM citric acid/Na phosphate buffer, 5 mM K_4_FE(CN)_6_ × 3 H_2_O, 5 mM K_3_FE(CN)_6_, 150 mM NaCl, 2 mM Mg/Cl_2_, 1 mg/ml X-Gal (AppliChem, Darmstadt, Germany), pH 6.0) for 48 h at 37 °C. Afterward, cells were washed three times with PBS for 30 s each and counterstained with Nuclear Fast Red (Sigma-Aldrich) according to the manufacturer’s protocol. Finally, cells were incubated in methanol for 1 min, air-dried, and cover-slipped.

### Cell viability assay

Cells were grown to 80% confluency and washed once with PBS. Afterward, they were incubated with PBS containing 5 mg/mL 3-(4,5-dimethylthiazol-2-yl)-2,5-diphenyltetrazolium bromide (MTT) (Sigma-Aldrich) for 30 min at 37 °C and 5% CO_2_. The supernatant was aspirated, and cells were lysed with MTT lysis buffer (0.12% HCl in isopropanol) for 10 min at RT. The same volume of ddH_2_O was added to each well, and the lysate was transferred to white 96-well OptiPlates (PerkinElmer, Waltham, MA). The absorption was measured at 590 nm using the GloMax^®^ Discover plate reader (Promega).

### RNA-seq library preparation and data quantification

RNA quality was determined with a Qubit^®^ (1.0) fluorometer (Life Technologies, Carlsbad, CA) and a fragment analyzer (Agilent, Santa Clara, CA). Only samples with a 260 nm/280 nm ratio between 1.8–2.1 and a 28S/18S ratio of 1.5–2 were further processed. The TruSeq Stranded mRNA kit (Illumina, Inc., San Diego, CA) was used for the following steps. Briefly, total RNA samples (100–1000 ng) were polyA enriched and then reverse-transcribed into double-stranded cDNA. The cDNA samples were fragmented, end-repaired and adenylated before ligation of TruSeq adapters containing unique dual indices (UDI) for multiplexing. Fragments containing TruSeq adapters on both ends were selectively enriched by PCR. The quality and quantity of the enriched libraries was validated using a Qubit^®^ (1.0) fluorometer and the fragment analyzer (Agilent). The product is a smear with an average fragment size of approximately 260 bp. The libraries were normalized to 10 nM cDNA in 10 mM Tris-HCl, pH8.5, with 0.1% Tween 20. The NovaseqX sequencer (Illumina, Inc.) was used for cluster generation and sequencing according to standard protocols. Sequencing was paired end at 2 × 150 bp. The RNA-seq data analysis consisted of the following steps: Raw reads were first cleaned by removing adapter sequences and poly-x sequences (>9 nt used for detection) using fastp (Version 0.20.0).^49^ Sequence pseudo alignment of the resulting high-quality reads to the Human Reference Genome (build GRCh38.p13) and quantification of gene expression level (gene model definition from GENCODE release 37) was carried out using Kallisto (Version 0.46.1).^50^ To detect differentially expressed genes we used the glm approach implemented in the software package DESeq2 (R version: 4.3.2, DESeq2 version: 1.42.0).^51^

### Analysis of published transcriptomic data

Transcriptomic data were acquired from the Gene Expression Omnibus (GEO) repository, accessing datasets GSE171170 and GSE202522. Subsequent differential expression analysis (DEA) was executed utilizing the BioJupies Plug-in library.^52^ Previously published skin single-cell RNA-seq data from human AD patients and healthy individuals^31^ were downloaded from GEO (GSE153760) as feature-barcode matrices (output of CellRanger v3.0.2) and further analyzed in R v4.3.2 with Seurat v5.0.1.^53^ We filtered out cells with less than 100 expressed genes and more than 10,000 expressed genes and cells with >60% ribosomal reads and >20% mitochondrial reads. Afterward, data were log normalized with 10,000 scale factor and scaled. To integrate data between samples, we used Harmony^54^ and further classified keratinocytes using *KRT14* and *KRT5* marker genes. To annotate keratinocyte subtypes in more detail we used data from^32^ and.^55^ To find differentially expressed genes between the conditions for all keratinocyte subtypes, we used the FindMarkers function from Seurat with default parameters.

### Gene set enrichment analysis (GSEA)

Microarray data from the epidermis of mice lacking Fgfr1 and Fgfr2 in keratinocytes vs control mice^18^ were re-analyzed using R v4.3.2 and *oligo* v1.66.0 and affycoretools v1.74.0 Bioconductor R packages. Differential expression was estimated using limma v3.58.1 and ranked gene sets by logFC were used for GSEA with clusterProfiler v4.10.1. Signature of genes significantly upregulated (FDR < 0.05) in lesional epidermis in comparison to normal epidermis were obtained from.^29^

### Statistical analysis

Statistical analysis and generation of graphs was performed using GraphPad Prism 10 software (GraphPad, Insight Partners, New York, NJ). The appropriate statistical tests were performed as indicated in the figure legends.

## Data Availability

All data are shown in Figures 1–7. Raw data will be provided by the corresponding author upon request. Original RNA-seq files are deposited in the Gene Expression Omnibus (GEO) (GSE267530) and are accessible via the following link: https://www.ncbi.nlm.nih.gov/geo/query/acc.cgi?acc=GSE267530
